# Clinical evaluation of prostate cancer gene 3 score in diagnosis among Chinese men with prostate cancer and benign prostatic hyperplasia

**DOI:** 10.1186/s12894-015-0110-x

**Published:** 2015-12-01

**Authors:** Jin Huang, Kathleen H. Reilly, Hui-Zhen Zhang, Hai-Bo Wang

**Affiliations:** Department of Pathology, Sixth People’s Hospital affiliated to Shanghai Jiaotong University, Yishan Rd 600#, Xuhui District, Shanghai, 200233 People’s Republic of China; New York City, NY USA; Peking University Clinical Research Institute, Xueyuan Rd 38#, Haidian District, Beijing, 100191 People’s Republic of China

**Keywords:** Prostate cancer, Prostate cancer gene 3, Prostate-specific antigen, China

## Abstract

**Background:**

Prostate cancer is the second most common diagnosed cancer in men. Due to the low specificity of current diagnosis methods for detecting prostate cancer, identification of new biomarkers is highly desirable. The study was conducted to determine the clinical utility of the prostate cancer gene 3 (PCA3) assay to predict biopsy-detected cancers in Chinese men.

**Methods:**

The study included men who had a biopsy at The Affiliated Sixth People’s Hospital of Shanghai Jiao Tong University from January 2013 to December 2013. Formalin-fixed, paraffin-embedded tissue blocks were used to test PCA3 and prostate-specific antigen (PSA) mRNA. The diagnostic accuracy of the PCA3 score for predicting a positive biopsy outcome was studied using sensitivity and specificity, and it was compared with PSA.

**Results:**

The probability of a positive biopsy increased with increasing PCA3 scores. The mean PCA3 score was significantly higher in men with prostate cancer (198.03, 95 % confidence interval [CI] 74.79–321.27) *vs* benign prostatic hyperplasia (BPH) (84.31, 95 % CI 6.47–162.15, *P* < 0.01). The PCA3 score (cutoff 35) had a sensitivity of 85.7 % and specificity of 62.5 %. Receiver operating characteristic analysis showed higher areas under the ROC curve for the PCA3 score vs PSA, but without statistical significance.

**Conclusions:**

Increased PCA3 in biopsy tissue correlated with prostate cancer and the PCA3 assay may improve the diagnosis efficacy as the PCA3 score being independent of PSA level. The diagnostic significance of urinary PCA3 testing should be explored in future study to determine the prediction value in guiding biopsy decision as the clinical relevance of current study was limited for PCA3 testing based on biopsy tissue in a limited number of Chinese men.

## Background

Prostate cancer is the second most common diagnosed cancer and the sixth leading cause of cancer deaths in men, accounting for 14 % of total new cancer cases [1]. Prostate cancer diagnosis primarily relies on prostate-specific antigen (PSA) and digital rectal examination (DRE) outcome. The presence of an abnormal DRE or an elevated PSA level is associated with increased risk of prostate cancer, which is followed by a biopsy [2]. The prostate cancer detection rate has greatly increased since the discovery of PSA and widespread PSA testing [3, 4]. Due to the low specificity of PSA, only 25–40 % of patients with a PSA of 2–10 ng/ml are diagnosed with prostate cancer on biopsy, resulting in a substantial number of unnecessary biopsies [5, 6]. Many patients experience pain, discomfort and anxiety following a biopsy, and unnecessary biopsies may lead to complications [7–9]. The identification of biomarkers capable of increasing the probability of a positive biopsy is highly desirable.

Recently the prostate cancer gene 3 (PCA3) has shown promise in identifying men at high probability of a positive biopsy and in guiding repeat biopsy decisions [10–15]. PCA3, which has been measured in urine in men at risk of prostate cancer, is over-expressed in prostate cancer cells compared with benign prostatic tissues [16]. Elevated PCA3 scores have been associated with a positive biopsy outcome [11], and the performance of PCA3 screening is maintained through repeat biopsies [10, 17]. In addition, PCA3 scores may be correlated with the tumor indexes of prostate cancer, such as tumor volume and Gleason score [18, 19].

Unfortunately, limited studies on the application of PCA3 scores in prostate cancer detection in the Chinese population are available in the literature [20]. Chinese men differ significantly from the Western population genetically. The clinical applicability of PCA3 scores in Chinese men should be investigated thoroughly. There was a high variability in cancer detection rates even among Asian populations, and Chinese-specific data is necessary to provide adequate information in counseling Chinese men who would consider prostate biopsy for suspected prostate cancer. Hence, the prostate biopsy database was set up in Department of Pathology, Sixth People’s Hospital affiliated to Shanghai Jiaotong University. The objective of the present study was to examine the performance characteristics of the PCA3 score in predicting biopsy-detected prostate cancer in the Chinese population. We also compared the performance of the PCA3 assay to that of PSA.

## Methods

### Study design

The study included men who had a biopsy at The Affiliated Sixth People’s Hospital of Shanghai Jiao Tong University from January 2013 to December 2013, excluding men with medical therapy known to affect PSA or invasive treatment for benign prostatic hyperplasia (BPH). The study was reviewed and approved by the Ethics Committee of The Affiliated Sixth People’s Hospital of Shanghai Jiao Tong University. All subjects received a detailed explanation of the study and written informed consent was obtained from all participants.

The histological slides were blinded and evaluated independently of the results of the other assays by two experienced pathologists. If the diagnosis differed between two pathologists, histological slides were reviewed again and a consensus diagnosis was obtained.

### Specimen collection and PCA3 assay procedure

Prostate cancer and BPH specimens were obtained from formalin-fixed, paraffin-embedded tissue blocks. We examined a series of 136 primary specimens collected by needle biopsy at the hospital. After adding 800 μl dimethylbenzene, tissue sections were incubated at 65 °C for 10 min to remove the paraffin. When tissue was to be manually dissected or scraped, the sections were immediately transferred to a microcentrifuge tube. The tube was centrifuged at 13,000 rpm for 3 min and the supernatant was sucked up. Then 800 μl 100 % ethanol was added and centrifuged again at 13,000 rpm for 5 min with the supernatant discarded. Then 800 μl 50 % ethanol was added and centrifuged at 14,000 rpm for 10 min with the supernatant discarded. Then 500 μL lysis mixture and proteinase K 5 μL were added to the centrifuge tube and incubated at 65 °C for 3 h. The sample tube was shaken at 1 h intervals during the incubation.

These samples after incubation were used for the detection of PCA3 and PSA mRNA according to the manufacturer’s instructions. The detection was based on branched DNA (bDNA) technology (DiaCarta, CA, USA), which is a sandwich nucleic acid hybridization procedure for the direct quantitation without RNA purification or reverse transcription polymerase chain reaction. The capture plate containing sample and bifunctional oligonucleotide probe sets was read on the Kodia QuantiVirus® Luminometer System by the supplied analysis software. The PCA3 score was calculated as (PCA3 mRNA)/(PSA mRNA) × 1000. Transrectal ultrasound guided biopsy with at least 10 peripheral zone cores was performed, and the specimens were reviewed by local pathologists.

### Statistical analyses

The age, values for the PCA3 scores, PSA levels, and % free PSA between prostate cancer patients and subjects with BPH were compared using the Wilcoxon rank sum test. In men with positive biopsy, the same test was used to examine the significance of differences in marker values from patients with Gleason scores ≤7 and with Gleason scores ≥8. Pearson correlation coefficients examined the relationship between PCA3 score and PSA level. Univariate associations of biopsy outcome with base predictors age, PSA and PCA3 score were evaluated using simple logistic regression analysis. In addition, adjusted odds ratios (aORs) were calculated for predictors that were statistically significant in the multivariate model.

The performance of PCA3 score for detecting prostate cancer was evaluated by sensitivity and specificity and their associated 95 % confidence intervals (CI) at various cutoff points using the receiver operating characteristic (ROC) analysis. The diagnostic accuracy of the PCA3 score was compared to that of PSA using the areas under the ROC curve (AUC) using the nonparametric method of Delong et al. [21]. The Youden index, calculated as sensitivity + specificity-1, was used for capturing the maximum vertical distance of the ROC curve and for determining cut-offs points. Statistical tests were performed using SAS 9.1 software (Cary,NC, USA). All tests were 2-tailed and *P* < 0.05 was considered the cut-off level for statistical significance for all analyses.

## Results

### Study population

Of the 136 included patients, 112 (82.4 %) had prostate cancer and 24 (17.6 %) were BPH on positive biopsy (Table 1). Ages ranged from 51 to 88 years and the median age was 70 years (interquartile range, IQR 66–77). Among the subjects with prostate cancer, the pathological biopsy Gleason score was ≤7 in 67.0 % and ≥8 in 33.0 % of men.

**Table 1:** The characteristics of the study population

	Prostate cancer	BPH^a^	*P* value
Age (Years)^b^	71.00 ± 11.00	67.00 ± 13.00	0.12
PSA(ng/ml)^b^	15.54 ± 39.21	8.70 ± 5.78	<0.01
PCA3 score	111.37 ± 278.26	17.76 ± 72.71	<0.01
<35	22 (19.6)	15 (62.5)	<0.01
≥35	90 (80.4)	9 (37.5)	
% free PSA^b^	0.12 ± 0.08	0.18 ± 0.17	0.03
Biopsy Gleason score			
≤7	75 (67.0)		
≥8	37 (33.0)		

^a^BPH, benign prostatic hyperplasia

^b^Median ± interquartile range

The probability of a positive biopsy increased with increasing PCA3 scores. The median PCA3 score was significantly higher in men with positive biopsy (111.37, IQR 42.64–320.90) vs BPH (17.76, IQR 9.19–81.90, *P* < 0.01). However, there was no significant difference in PCA3 scores between prostate cancer patients with biopsy Gleason score ≤7 (113.68, IQR 49.38–326.44) and patients with biopsy Gleason score ≥8 (102.59, IQR 12.07–278.23, *P* = 0.27). There was also no significant difference in PCA3 scores between prostate cancer patients with biopsy Gleason score ≤6 (94.26, IQR 41.81–326.44) and patients with biopsy Gleason score ≥7 (139.02, IQR 42.66–295.69, *P* = 0.56).

The probability of a positive biopsy also increased with increasing PSA. The median PSA was 13.67 (IQR, 7.98–29.02), and the prostate cancer patients had a significantly higher median PSA level (15.54, IQR 8.49–47.70) than BPH subjects (8.70, IQR 6.39–12.17, *P* < 0.01). The prostate cancer patients with Gleason score ≥8 had a significantly higher PSA level (54.09, IQR 13.46–100.00) than those with Gleason score ≤7 (13.21, IQR 8.43–21.25, *P* < 0.01). The PSA was <4 ng/ml in 9 men (6.6 %), 4–10 ng/ml in 37 men (27.2 %) and ≥10 ng/ml in 90 men (66.2 %). There was no significant difference in PCA3 scores among 3 groups of subjects with different PSA level. No relationship was found between PCA3 score and PSA based on correlation analysis (r = 0.08, *P* = 0.33).

### ROC curve

**Fig. 1:**
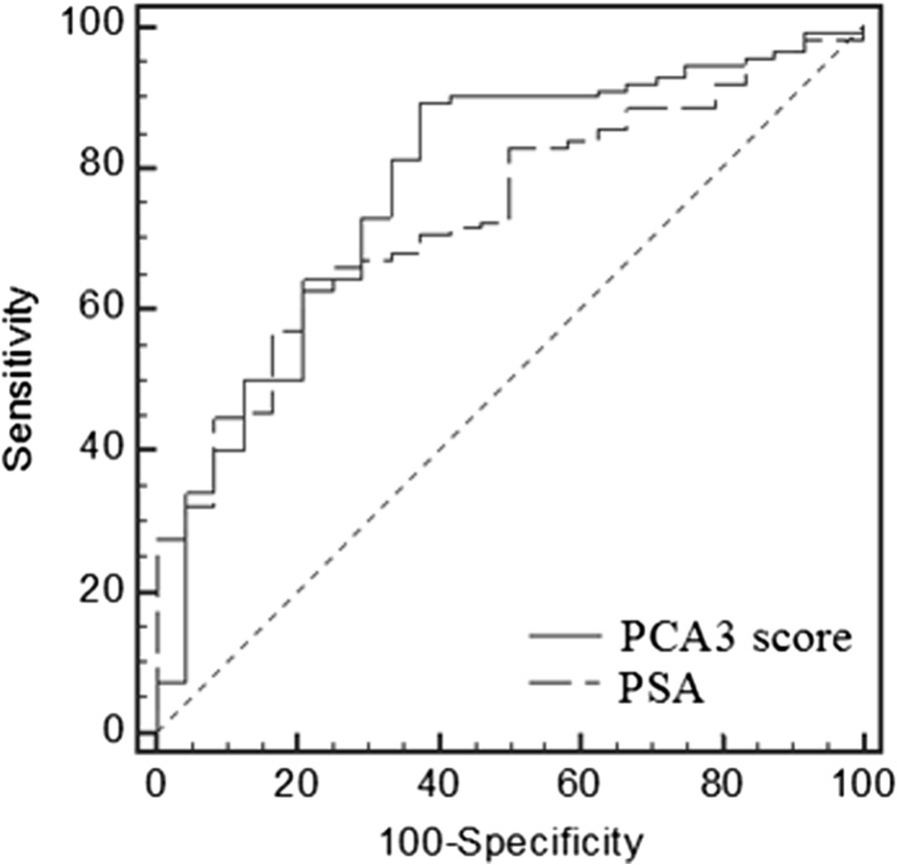
ROC curve of PCA3 score and PSA for detecting prostate cancer. PCA3: prostate cancer gene 3; PSA: prostate-specific antigen. ROC: receiver operating characteristic

A ROC curve was used to demonstrate the diagnostic performance of PCA3 score and PSA for detecting prostate cancer (Fig. 1). The figure included the sensitivity and specificity of the two assays. The AUC of PCA3 score and PSA was 0.775 (95 % CI, 0.695–0.842) and 0.736 (95 % CI, 0.653–0.808), respectively. ROC analysis showed that there was no significant difference in AUC between PCA3 score *vs* PSA (*P* = 0.60).

### Correlation of PCA3 score and PSA with diagnosis of prostate cancer

Results in terms of sensitivity and specificity are shown in Table 2. The PCA3 score cutoff of 35 provided the optimal balance (i.e., the maximum sum of sensitivity and specificity) between sensitivity (80.4 %) and specificity (62.5 %). Subjects with a PCA3 score of 35 or greater had a 6.8-fold higher probability of a positive biopsy than those with a PCA3 score less than 35 (*P* < 0.01) (Table 3).

**Table 2:** Sensitivity and specificity of the PCA3 score and PSA

	% Sensitivity (95 % CI)	% Specificity (95 % CI)
PCA3 score cutoff 20	83.0 (74.8–89.5)	58.3 (36.6–77.9)
PCA3 score cutoff 35	80.4 (71.8–87.3)	62.5 (40.6–81.2)
PSA cutoff 4	95.5 (89.9–98.5)	16.7 (4.7–37.4)
PSA cutoff 10	71.4 (62.1–79.6)	58.3 (36.6–77.9)

**Table 3:** Univariate and multivariate logistic regression analyses in predicting prostate cancer detection upon prostate biopsy

	OR (95 % CI)	*P* value
Univariate logistic regression model		
Age	1.05 (0.99–1.11)	0.14
% free PSA	1.22 (0.75–1.98)	0.43
PSA level (ng/ml)		
<4	1.0	
4 ~ 10	2.16 (0.48–9.70)	0.31
>10	6.40 (1.47–27.83)	0.01
PCA3 score		
<35	1.0	
≥35	6.82 (2.64–17.61)	<0.01
Multivariate logistic regression model		
PSA level 4 ~ 10 ng/ml	2.66 (0.50–14.08)	0.25
PSA level >10 ng/ml	6.99 (1.38–35.33)	0.02
PCA3 score ≥35	6.73 (2.49–18.14)	<0.01

The sensitivity of PCA3 score (≥35) was 80.4 % (90/112) (95 % CI 71.8–87.3) and the specificity was 62.5 % (15/24) (95 % CI, 40.6–81.2). The sensitivity and specificity of PSA (≥4 ng/ml) were 95.5 % (107/112) (95 % CI 89.9–98.5) and 16.7 % (4/24) (95 % CI 4.7–37.4), respectively; while the sensitivity and specificity of PSA (≥10 ng/ml) were 71.4 % (80/112) (95 % CI 62.1–79.6) and 58.3 % (14/24) (95 % CI 36.6–77.9). The sensitivity for detecting prostate cancer was comparable, but the specificity was significantly lower for PSA (≥4 ng/ml) than PCA3 score (≥35). Although without statistical significance, both sensitivity and specificity of PSA (≥10 ng/ml) were lower than PCA3 score (≥35).

### Univariate and multivariate logistic regression analyses

In univariate analysis, PSA >10 ng/ml (vs. <4 ng/ml) and a PCA3 score of ≥35 were significant independent predictors of positive biopsies (Table 3). In multivariate analysis, a PCA3 score of ≥35 remained significant independent predictor of positive biopsies (adjusted OR 6.73; 95 % CI 2.49–18.14). PSA >10 ng/ml (vs. <4 ng/ml) was also significant (*P* = 0.02).

## Discussion

Due to the low specificity of PSA for detecting prostate cancer, unnecessary biopsy remains substantial [5]. The discovery and the development of novel biomarkers for prostate cancer diagnosis remains a challenge, despite the widespread use of PSA and DRE. The study evaluated the PCA3 assay as an additional tool in facilitating diagnosis of prostate cancer in Chinese men. An increasing PCA3 score corresponded with an increasing probability of a positive biopsy. The mean PCA3 score was significantly higher in men with positive biopsy *vs* a negative biopsy. The slight superiority of diagnostic accuracy of PCA3 score over PSA level was shown in this study, although without statistical significance. Data was also consistent with the PCA3 score being independent of PSA level [10, 11].

In a European multicenter study, the diagnostic accuracy of the PCA3 score was evaluated in men undergoing an initial biopsy [22]. The AUC ROC for the PCA3 score for predicting biopsy outcome was 0.761 and comparable to that in this study at 0.775. In European PCA3 studies, the AUC ROC was 0.761 in the initial and 0.658 in the repeat biopsy study [10]. These results suggested that the PCA3 assay can be used to guide both initial and repeat biopsy decisions. Therefore, PCA3 score may be considered clinically meaningful and its application in clinical practice can be justified [22, 23]. The PCA3 score cutoff of 35 provided the optimal balance between sensitivity (80.4 %) and specificity (62.5 %). Subjects with a PCA3 score of 35 or greater had a 6.8-fold higher probability of a positive biopsy than those with a PCA3 score less than 35 (*P* < 0.01). However, the additive value of PCA3 score in predicting biopsy outcome and the most optimal PCA3 score cutoff should be further evaluated by prospective studies to identify men with a high probability of a positive biopsy.

Multiple studies have also compared the diagnostic performance of the PCA3 assay to that of the traditional biomarkers, such as PSA and % free PSA [10, 11, 19]. These studies have shown the superiority of the PCA3 score over PSA level, although with slight improvement [19]. In our study, the AUC of PCA3 score and PSA was 0.775 (95 % CI, 0.695–0.842) and 0.736 (95 % CI, 0.653–0.808), respectively; and there was no significant difference (*P* = 0.60). This discrepancy can be explained by the smaller sample of men and by the restricted proportion of negative biopsy studied in the present report (82.4 % positive biopsy); this resulted in a decrease of statistical power. However, the sensitivity for detecting prostate cancer was comparable, but the specificity was significantly lower for PSA (≥4 ng/ml) than PCA3 score (≥35); both sensitivity and specificity of PSA (≥10 ng/ml) were lower than PCA3 score (≥35), although without statistical significance. Most importantly, data were also consistent with the PCA3 score being independent of PSA level, i.e., the diagnostic accuracy of the PCA3 score was not affected by PSA levels, confirming the findings of prior studies [10, 11, 22]. It was demonstrated that PCA3 fulfilled the most stringent criteria for a novel marker, i.e., in addition to univariate discriminatory ability it improved sensitivity and specificity and confirmed its independent predictor status [23].

In the analysis of the overall cohort of the European study, Haese et al. found that the PCA3 score was significantly higher in men with high Gleason scores [10]. Studies evaluating men undergoing radical prostatectomy showed an association between PCA3 score, tumor volume and Gleason score [24]. Our findings did not confirm this. This discrepancy can be explained by the smaller sample of men. Men at higher risk of aggressive and high Gleason score prostate cancer were studied in the present study (33.0 % patients with Gleason score ≥8). This resulted in a decrease of statistical power. However, other studies also questioned the relationship between the PCA3 score and aggressive prostate cancer [10, 11, 18, 19]. The association between PCA3 score and aggressive prostate cancer needs further evaluation in controlled studies to confirm the utility in selecting men with clinically insignificant prostate cancer.

The current study was subject to several limitations. The study population was referred to a PCA3 test for several reasons (i.e., a high PSA level or suspicious prostate cancer), therefore, those who were selected to have a PCA3 test because of a clinical concern for prostate cancer may differ from screening populations referred to triage testing. These subjects in the study had a median age of 70 years (IQR 66–77) with a relatively high PSA level (median 13.67; IQR 7.98–29.02), which is higher than that of a typical screening population [15]. The subjects recruited with high clinical suspicion for prostate cancer could not represent the population in China, more unlikely to reflect the actual situation in China. Secondly, the study sample was relatively small (especially the number of participants with negative biopsy) to compare the clinical performance of PCA3 score and serum PSA testing. Finally, PCA3 testing was based on formalin-fixed, paraffin-embedded tissue blocks collected before biopsy, therefore the clinical relevance was limited. To help determine the need for biopsy decision in screening populations, the diagnostic significance of urinary PCA3 testing will be explored in a future study.

## Conclusions

This study showed that increased PCA3 in biopsy tissue correlated with prostate cancer and that the PCA3 assay could aid in diagnosis of prostate cancer in a limited number of Chinese men. The probability of a positive biopsy increased with increasing PCA3 score. In this population, the PCA3 score had a comparable diagnostic accuracy with PSA as there was no significant difference in ROC AUC between PCA3 score and PSA. Most importantly, the PCA3 assay confirmed its independent diagnosis value and may improve the diagnosis efficacy as the PCA3 score being independent of PSA level. However, the clinical relevance was limited as PCA3 testing was based on biopsy tissue. To help determine the prediction value in guiding biopsy decision, the diagnostic significance of urinary PCA3 testing should be explored in future study.

## Abbreviations

PSA: Prostate-specific antigen
DRE: Digital rectal examination
PCA3: Prostate cancer gene 3
BPH: Benign prostatic hyperplasia
aORs: Adjusted odds ratios
CI: Confidence intervals
ROC: Receiver operating characteristic
IQR: Interquartile range
